# Healthcare Capacity, Health Expenditure, and Civil Society as Predictors of COVID-19 Case Fatalities: A Global Analysis

**DOI:** 10.3389/fpubh.2020.00347

**Published:** 2020-07-03

**Authors:** Jahidur Rahman Khan, Nabil Awan, Md. Mazharul Islam, Olav Muurlink

**Affiliations:** ^1^Health Research Institute, Faculty of Health, University of Canberra, Bruce, ACT, Australia; ^2^Biomedical Research Foundation, Dhaka, Bangladesh; ^3^Institute of Statistical Research and Training, University of Dhaka, Dhaka, Bangladesh; ^4^Department of Biostatistics, University of Pittsburgh, Pittsburgh, PA, United States; ^5^Bangladesh Institute of Governance and Management (BIGM), Dhaka, Bangladesh; ^6^School of Business and Law, Central Queensland University, Brisbane, QLD, Australia

**Keywords:** COVID-19, healthcare capacity, population density, healthcare expenditure, civil society

## Abstract

**Background:** The rapid growth in cases of COVID-19 has challenged national healthcare capacity, testing systems at an advanced ICU, and public health infrastructure level. This global study evaluates the association between multi-factorial healthcare capacity and case fatality of COVID-19 patients by adjusting for demographic, health expenditure, population density, and prior burden of non-communicable disease. It also explores the impact of government relationships with civil society as a predictor of infection and mortality rates.

**Methods:** Data were extracted from the Johns Hopkins University database, World Bank records and the National Civic Space Ratings 2020 database. This study used data from 86 countries which had at least 1,000 confirmed cases on 30th April 2020. Negative binomial regression model was used to assess the association between case fatality (a ratio of total number of confirmed deaths to total number of confirmed cases) and healthcare capacity index adjusting for other covariates.

**Findings:** Regression analysis shows that greater healthcare capacity was related to lesser case-fatality [incidence rate ratio (IRR) 0.5811; 95% confidence interval (CI) 0.4727–0.7184; *p* < 0.001] with every additional unit increase in the healthcare capacity index associated with a 42% decrease in the case fatality. Health expenditure and civil society variables did not reach statistical significance but were positively associated with case fatalities.

**Interpretation:** Based on preliminary data, this research suggests that building effective multidimensional healthcare capacity is the most promising means to mitigate future case fatalities. The data also suggests that government's ability to implement public health measures to a degree determines mortality outcomes.

## Introduction

The novel coronavirus 2019 (COVID-19) was classified by the World Health Organisation as a new virus on the 12th of January 2020, declared a pandemic on the 11th of March, and by the end of the following month was present in over 200 nations, with 3 million cases and over 200,000 deaths (1). This rapid escalation has been attributed to a range of factors including virulence and rapid asymptomatic human-to-human transmission (2) accelerated through the medium of global air travel (3). While fatality rates are still being estimated using incomplete data, of those diagnosed with COVID-19 the majority recover without hospitalization, with ~20% of the patients hospitalised with severe breathing difficulties (4). The speed with which the pandemic has taken hold, has limited preparation time for national health systems, and the requirement for high care hospitalisation associated with the disease, far in excess of normal capacity in national health systems, represents a major new health challenge.

Patients requiring treatment may require advanced ICU procedures, including bronchoscopy, percutaneous tracheostomy, and extracorporeal membrane oxygenation (ECMO) (5), but in addition to advanced treatment, ICU equipment and intensive care beds, the pandemic has also dramatically escalated demand for relatively low-technology but high-quality personal protective equipment (PPE) even within advanced economies. Early warning of the extraordinary short-term strain the disease could pose to health systems came from the origin city of Wuhan in China, where the government rushed to build vast temporary facilities to cope with the explosion in infections (6).

Regional and national responses to the pandemic have differed. South Korea acted early, monitoring people entering Korea from Wuhan in December, and using intensive tracking of known cases and testing of their contacts, and creating discrete COVID-19 and non-COVID-19 health sectors to ensure treatment of non-virus patients could continue during the pandemic (7). With Europe declared an epicentre of COVID-19, the European Centre for Disease Prevention and Control attempted to introduce a co-ordinated management guideline and introduced contact tracking technology across the European Union (EU) countries to prevent secondary infection (8, 9). Most of the EU countries responded by closing borders with contiguous countries, imposing a travel ban and restricting the export of protective and medical equipment (8), but the European response was not unified, with officials in some member states openly considering “herd immunity.” Germany, a developed EU country which ranked fourth in world nominal GDP and contributing 28% of the Euro-based economy, expanded its ICU capacity from 28,000 to 40,000 beds and instituted strict controls on social contact within its borders (1, 10). The United States (US), like Europe, experienced a relatively disintegrated response to the crisis, partly as result of conflict between federal responses and individual states administrations' responses (11), however, with the US spending almost twice as much *per capita* on medical care than other high-income countries (12) scrutiny on that nation's “maximum-possible-test-per-day” strategy and utilisation of its 96,596 ICU beds (13) has been particularly intense.

In developing countries, the picture has been significantly less clear. Most South Asian countries introducing restrictions on international air travel, countrywide lockdowns, and declaring a general vacation in workplaces and educational institutions (14), in many cases doing so earlier than the developed world. Public health, as opposed to medical responses, has been the emphasis. The medical response, ranging from testing to treatment, has been relatively muted. With lower per-capita spending on health, and a higher underlying burden of disease and higher population densities, developing nations' healthcare systems face a combination of increased threat and a reduced healthcare capacity to respond. They have much lower ratios of ICU beds and advanced equipment per population, and fewer medical staff. For example, Bangladesh, with a population of around 165 million, has fewer than seven thousand beds in isolation units and about just 1622 health professionals comprising only 595 doctors for treating COVID-19 patients (15) however, the nation has a highly advanced public health care system experienced at dealing with infectious disease (16).

This paper analyses a number of factors regarded as plausibly significant in determining COVID-19 fatality globally, including healthcare expenditure *per capita* and population density. Healthcare expenditure in particular, is highly variable. There are other healthcare variables known to sensitively predict mortality rates independent of expenditure per capita or as a proportion of gross domestic product (GDP). Robinson et al. (17) analysed the global distribution of health professionals, both physicians and nurses, in a regression analysis controlling for GDP, finding that the proportion of physicians in particular are related to infant and child (under-5) mortality rates globally, and countries with a high proportion of medical professions relative to their GDP showing unusually low mortality rates.

This study aims to assess the association between the healthcare capacity and the case-fatality of COVID-19 patients by adjusting for health expenditure as a percentage of GDP, population density and two variables that capture health vulnerability prior to the pandemic, the proportion of the population over 65 years old, and the burden of non-communicable disease (NCD). Finally, this study examines a variable rarely used in epidemiological studies, one that plausibly explains the degree to which a government can enact public health measures in the face of the crisis. The CIVICUS Civil Society Index broadly estimates the degree to which governments control their citizenry and the citizenry in turn influence the government. In a public health response highly reliant on controlling the freedom of movement of the public, high degrees of customary government control should, *a priori*, be related to lower levels of COVID-19 outbreaks.

## Methods

### Data Sources

#### COVID-19 Cases and Deaths

Data about the cumulative number of confirmed COVID-19 cases and deaths were extracted from the Johns Hopkins University (JHU) database on 30th April 2020 (18).

#### COVID-19 Tests

Data on the number of tests by country were extracted from Worldometer on 30th April 2020 (19). The Worldometer figures are based on national Ministries of Health and other government authorities, including at a local government level (20).

All other variables, other than the civil society data, were drawn from the latest estimates (not always the same year) from World Bank records (21).

#### Non-communicable Diseases (NCD)

Non-communicable diseases (NCD) are representative of the NCD-related death burden [cause of death, by NCDs (percentage of total)], which in the following analysis acts a proxy for which populations are more prone to COVID 19-related death based on ample evidence that comorbidity is a key predictor of health outcomes for those infected with the virus (22).

#### Healthcare Capacity

The number of nurses and midwives per 1,000 people, the numbers of physicians per 1,000 people and the numbers of hospital beds per 1,000 people, were included in the analysis.

#### Civil Society

The CIVICUS Civil Society Index (CSI) (23) is based on interviews with both key stakeholders and citizenry on questions related to the state of civil society. The developers of the index define civil society as “the arena, outside of the family, the state, and the market where people associate to advance common interests” (p 378). The index is composed of four dimensions, namely structure (relating to the size and composition of nation's civil society), environment (political, legal, institutional, social and cultural factors, as well as attitude of private and state sector actors toward the nation's civil sector) values (being the degree to which civil society actors tolerance toward other actors, as well as other values, for example toward the environment) and finally impact (relating to civil societies' interface with the world of governance and policy). The CSI gives rise to a categorical ranking, with societies accorded either “open,” “narrowed,” “obstructed,” “repressed,” or “closed” appellation. While the index is designed to capture a broad notion of civil society, as the labels suggest, there is a close relationship between the index and the constructs of civil *freedom* as well as civil *respect*. At one extreme, lies the “open society” which CIVICUS state sees citizens “free to form associations, demonstrate in public places and receive and impart information without restrictions in law or practice” (freedom), and “authorities are tolerant of criticism from civil society groups and provide space and platforms for open and robust dialogue” (respect). At the other extreme, a “closed society” is characterised as one where “state and powerful non-state actors are routinely allowed to imprison, seriously injure and kill people with impunity for attempting to exercise their rights” (freedom) and “criticism of the ruling authorities is severely punished and there is virtually no media freedom” (respect) (24). Civil society rating data were extracted from the National Civic Space Ratings 2020 database (25).

#### Other Variables

Current health expenditure as a percentage of GDP, population density people per square kilometre (km) of land area, and proportion of population aged 65 or above were drawn from World Bank data.

## Analytic Sample and Statistical Analysis

In this study, countries with at least 1,000 confirmed COVID-19 cases (*n* = 86) were included. Moreover, the number of tests data were available for 83 countries except for China, Cameroon, and Guinea. Therefore, the sample size for main analysis was *n* = 86 countries, where sample size for sub-analysis was *n* = 83 countries.

Based on the healthcare capacity variables, a single index was calculated using principal component analysis (PCA), which has widely been used in health research to construct indexes. This method presents the original high dimensional data in a new coordinate system as linear combinations of original variables that capture the common information most efficiently. These linear combinations are called principal components (PC), where the first PC explains the largest amount of variation in the original data, and the last PC explains the least of amount of variation. Method of creating weights from the results of PCA is to assume that the first PC corresponds to the underlying process which the index is trying to measure. An index is then measured by calculating a score for each observation consisting of the sum of the variable values multiplied by the calculated weights. Health capacity index was categorised into three classes using tertiles (and labelled “low,” “middle,” “high”). In addition, health expenditure as a percentage of GDP was also categorised into tertiles (with the same descriptors).

Descriptive statistics (median, 25th and 75th quantiles) were calculated for all variables analysed in this study. Boxplot and scatter plot [with locally estimated scatterplot smoothing (LOESS)] were used to perform empirical analysis for some key variables.

Negative binomial (NB) regression model was employed to assess the association between outcome and predictor/covariates. The dependent variable was specified as the count of deaths. The NB model was adjusted for the number of confirmed cases to consider case fatality (defined as the ratio of total number of confirmed deaths to total number of confirmed cases) by incorporating the logarithm of the number of confirmed cases as an offset term. The main predictor variable was the healthcare capacity index/class, and the outcome variable was the number of deaths. A set of covariates were adjusted in the model including population density (logarithm scale), proportion of population ages 65 or above, health expenditure, non-communicable disease-related deaths and civil society classes. The statistical software *R* (version 3.6.3) was used for analysing data (26). In regression analysis, the level of significance was set at α = 0.01.

## Results

The exploratory analysis demonstrated that the median number of confirmed cases and deaths among 86 countries (see Appendix 1 in Supplementary Material) during the period analysed was ~7,320 and 199 (see Table 1). This number of course represents a single point in time. Median population density per square km was 86.59, and median percentage of population aging 65 and above was 11.72. Median current health expenditure as a percentage of GDP was 6.84. Median percentage of NCD-related deaths was 85.75. Among these countries, median number of nurses and midwives, physicians, and hospital beds per 1,000 people was 5.80, 2.85, and 2.44, respectively (see Table 1).

**Table 1:** Summary statistics of selected variables (*n* = 86).

**Variables**	**Median**	**1st quantile to 3rd quantile**
Confirmed	7,320	2,106–20,265
Deaths	199	61–873
Population density^a^	86.59	45.24–145.33
Population ages 65 and above^b^	11.72	6.18–18.38
Current health expenditure^c^	6.84	5.03–8.98
NCD-related deaths^d^	85.75	73.38–89.80
Nurses and midwives per 1,000 people	5.80	2.07–8.60
Physicians per 1,000 people	2.85	1.60–5·00
Hospital beds per 1,000 people	2.44	1.34–3.42
Healthcare capacity index	0.22	−1.44 to 1.14

a *Population density (people per square km of land area)*.

b *Population ages 65 and above (% of total population)*.

c *Current health expenditure as a percentage of GDP*.

d *Cause of death, by non-communicable diseases (percentage of total)*.

Two models, one for the continuous healthcare capacity index (Model 1) and another one for the categorised index (Model 2) adjusting for other factors were fitted (Table 2). The Nagelkerke *R*^2^ was higher for Model 1 (*R*^2^: 0.578) compared to the Model 2 (*R*^2^: 0.484).

**Table 2:** Estimated association between healthcare capacity and deaths adjusting for other covariates (*n* = 86).

**Variables**	**Model 1**	**Model 2**
	**IRR (95% CI)**	**IRR (95% CI)**
Intercept	0.0007 (0.0001–0.0039)^a^	0.0021 (0.0004–0.0115)^a^
Population density (log)^1^	1.0749 (0.9299–1.2409)	1.1090 (0.9492–1.2924)
Population ages 65 and above^2^	1.1010 (1.0381–1.1684)^a^	1.0754 (1.0123 to 1.1433)^b^
Healthcare capacity index (continuous)	0.5811 (0.4727–0.7184)^a^	
Healthcare capacity index (reference: low)		1.0000
Middle		0.4682 (0.2703–0.8087)^b^
High		0.2846 (0.1440–0.5646)^a^
Current health expenditure^3^	1.1804 (1.0818–1.2917)^a^	1.1346 (1.0403–1.2427)^b^
NCD-related deaths^4^	1.0165 (0.9987–1.0337)	1.0162 (0.9969–1.0350)
Civil society (reference: open)		
Narrowed	0.8050 (0.5072–1.2693)	0.8471 (0.5035–1.4237)
Obstructed	1.0967 (0.6051–2.0133)	1.1634 (0.6177–2.2290)
Repressed	1.3378 (0.6966–2.5872)	1.4645 (0.7157–3.0250)
Closed	0.8878 (0.4424–1.8276)	0.8591 (0.3995–1.9051)
*R*^2^ Nagelkerke	0.578	0.484
AIC	1164.220	1175.443

*IRR, incidence rate ratio; CI, confidence interval; p-value: ^b^p < 0.01; ^a^p < 0·001; AIC, Akaike information criteria*.

1 *Population density (people per square km of land area)*.

2 *Population ages 65 and above (% of total population)*.

3 *Current health expenditure as a % of GDP*.

4 *Cause of death, by non-communicable diseases (percentage of total)*.

Case fatality due to COVID-19 was associated negatively with health capacity index (IRR 0.5811; 95% CI 0.4727–0.7184; *p* < 0.001), with a 1% increase in the healthcare capacity index associated with a striking 42% decrease in the case fatality. By contrast, greater health expenditure as a percentage of GDP was positively associated with case fatality (IRR 1.1804; 95% CI 1.0818–1.2917; *p* < 0.001). The proportion of the population aged over 65 years was positively related to case fatality (IRR 1.1010; 95% CI 1.0381–1.1684; *p* < 0.001).

The model with categorised health capacity index confirmed that countries with middle and high health capacity were inversely associated with case fatality (Table 2). Other variables revealed a similar direction of associations.

**Figure 1:**
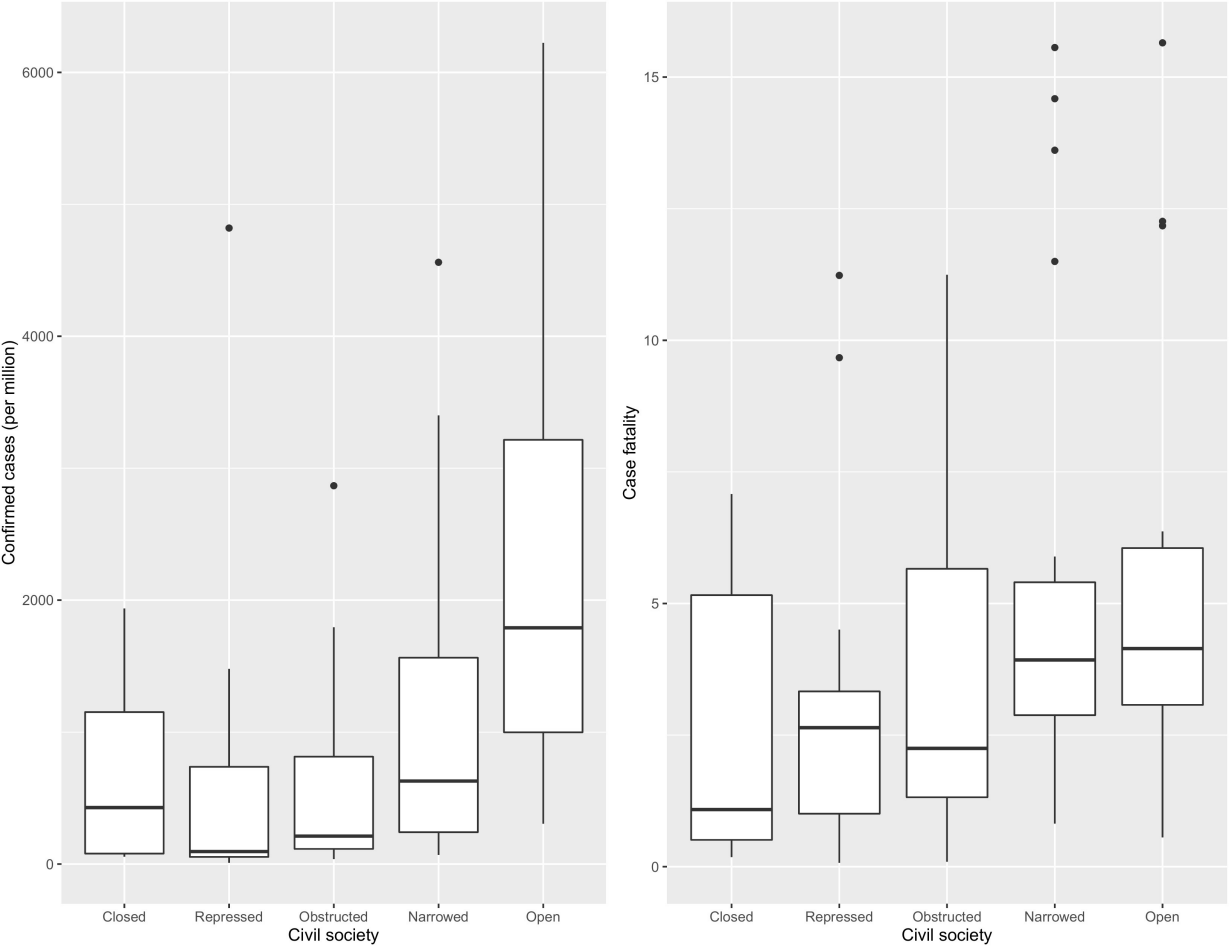
Civil society wise distribution of the number of cases per million and case fatality.

Amongst the countries included in this study, 29.07% were categorised as “narrowed,” followed by “open” (20.93), “repressed” (19.77), “obstructed” (18.60), and “closed” (11.63). Figure 1 shows confirmed cases per million and case fatality varied across different categories of CSI. The highest number of confirmed cases per million were found in countries with an “open” civil society and the lowest in countries with a repressed civil society. On the other hand, case fatality was highest in countries with a narrowed civil society followed by an open, where the lowest case fatality was observed in countries with a closed civil society. Thus, closed civil societies were associated with the lowest number of cases and fatalities.

**Figure 2:**
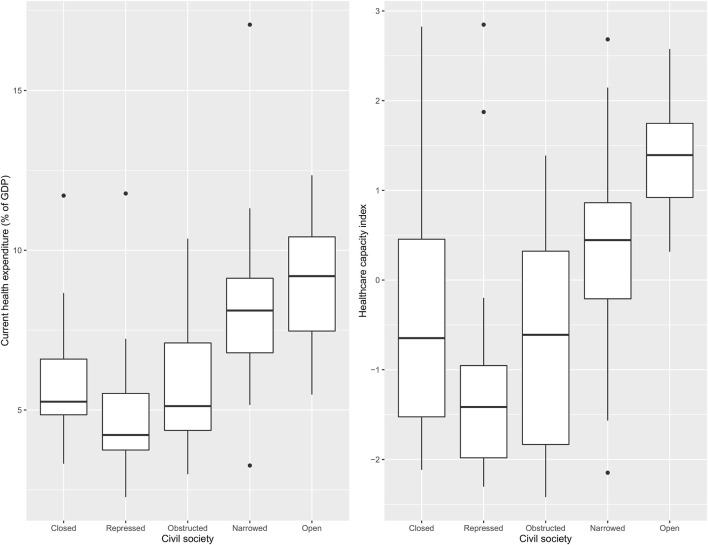
Civil society wise distribution of health expenditure and health capacity index.

Figure 2 shows current health expenditure as a percentage of GDP and a healthcare capacity index value across different categories of CSI. The highest value in both indicators were found in countries with an “open” civil society and the lowest in countries at the opposite end of the CSI spectrum, “repressed” civil societies.

**Figure 3:**
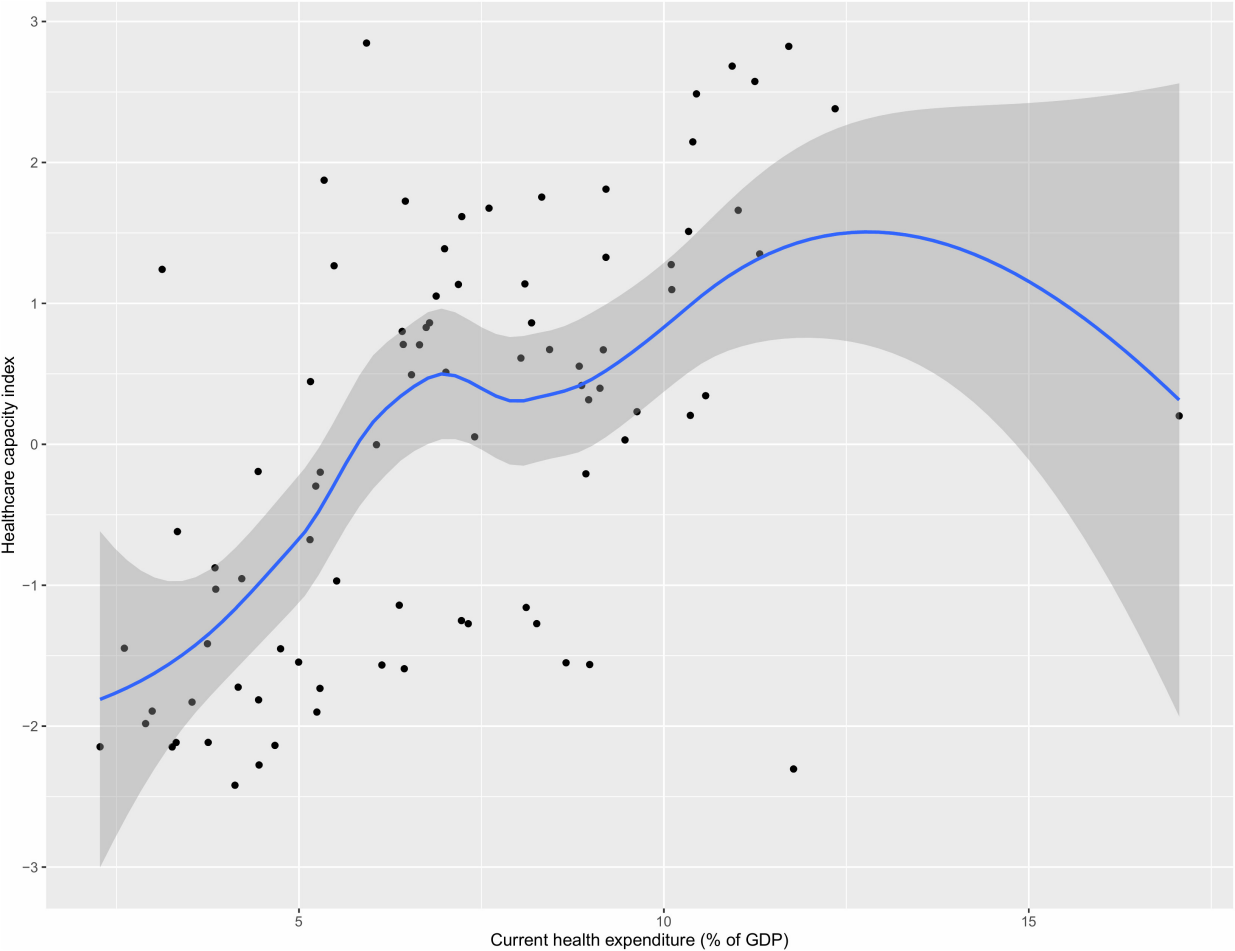
Health expenditure and health capacity index (LOESS) plot.

Figure 3 shows the relationship between current health expenditure as percentage of GDP and healthcare capacity index. The figure shows a positive relationship, with greater health expenditure associated with greater healthcare capacity index in general, with a few exceptions where some countries with higher health expenditure (as their % of GDP) did not score correspondingly high in terms of healthcare capacity such as USA (highest % expenditure but medium healthcare index), and Afghanistan (high % expenditure but low healthcare index).

**Figure 4:**
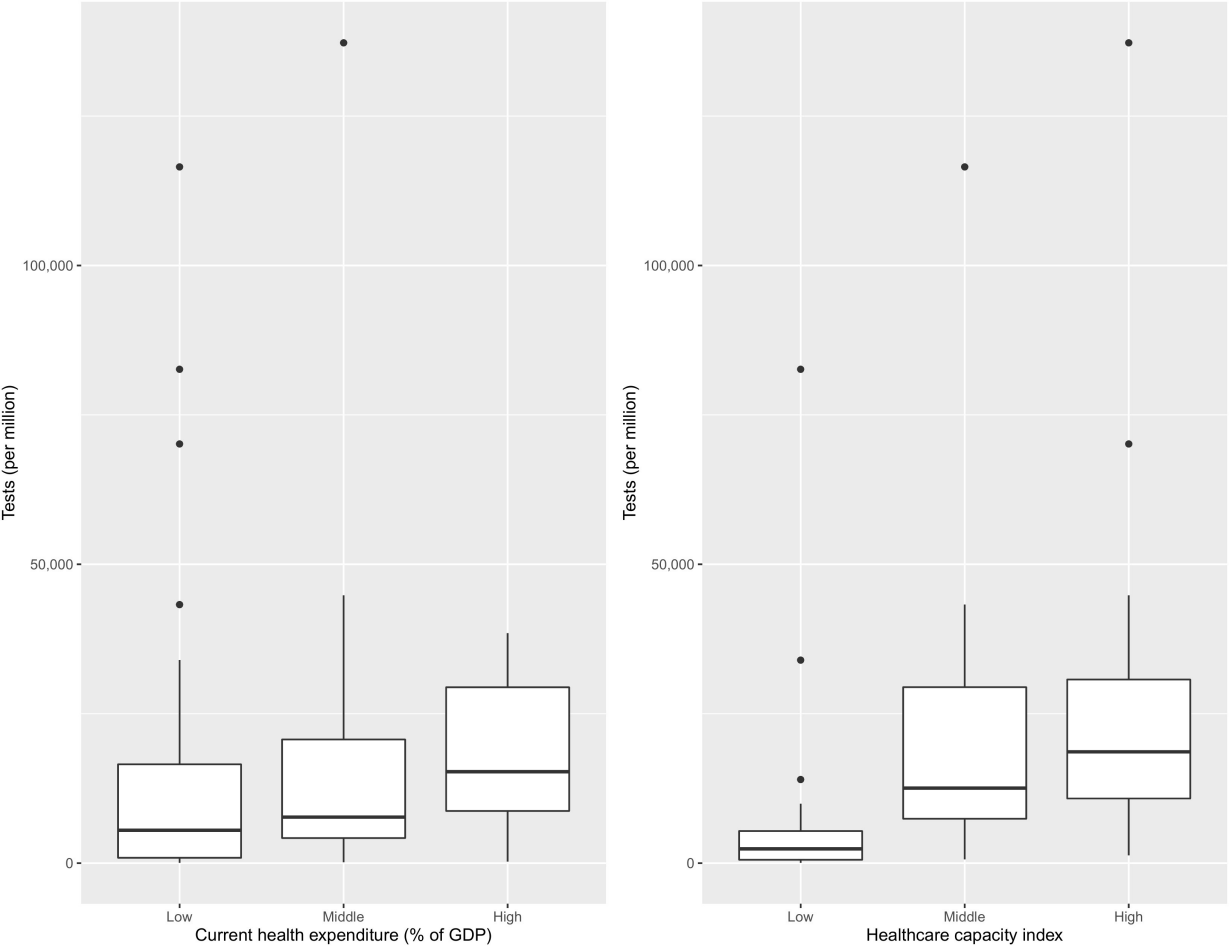
Health expenditure and healthcare capacity group wise distribution of the number of tests per million (*n* = 83).

Figure 4 shows the number of tests per million population across clusters of nations with differing healthcare capacity and health expenditures. Countries with higher healthcare capacity clearly had higher testing rates. The difference was less pronounced as a function of health expenditure.

## Discussion

This study shows that controlling for factors such as expenditure on health care, the existing burden of non-communicable disease, the age profile of the population and population density, greater healthcare capacity is sensitively related to lesser COVID-19 case-fatality, with each additional unit increase in the healthcare capacity index associated with a 42% decrease in case fatalities. In addition it points to an association between the strength of civil society and an ability to enact adequate public health responses to pandemic such as COVID-19. This study has taken place against the background of an explosion in scholarly interest in the COVID-19 pandemic since the emergence of the novel coronavirus with studies examining the role of asymptomatic carrier transmission (27), pathological and pathogenetic associates with acute respiratory syndrome (28), and the role of demographic risk factors (such as gender, age, and comorbidities) in predicting virulence (29, 30).

This current analysis adds to a group of studies that have modelled how mitigation measures may change the course of the disease in different countries (31–35). While there has been some discussion on the relationship between mortality and healthcare resource availability in different regions and provinces of China (36), there has been limited research in this vein, in part because the crisis is still evolving, and there is a great deal of uncertainty at a clinical level, as well as at a cruder actual level of understanding what capacity exists and what relevant factors to include in capacity and vulnerability studies (37). For example, a US-focused study has explored how equitable allocation of resources can buttress a nation's capacity to defend itself against the pandemic (38), but it appears that even highly-developed nations have failed to take long-term measures to strengthen healthcare systems to deal with the fluctuations in demand posed by a pandemic.

The finding that countries with strong healthcare capacity had fewer deaths per confirmed case was unsurprising. In this sense, this study confirms previous research on the association of overall mortality (all causes) and healthcare (39). Likewise, higher share of 65+ population being associated with higher fatality was also anticipated, as COVID-19 has been more fatal for the elderly (40). This variable acted as a confound for the share of NCD-related death burden, which was independently associated with COVID-19 fatality, again confirming existing research showing the role of comorbidity in determining poor clinical outcomes (41). With older age related to higher rates of NCD (42), this confound was expected.

More surprisingly, in the regression analysis higher population density was not associated with higher fatality in countries that met inclusion criteria. It is commonly assumed that population density increases the risk of transmission of COVID-19 (43). But early lockdown and social distancing measures, particularly in nations with lower GDP and higher control over their citizenry may have subdued the impact of density. While the CSI did not reach significance in the regression analysis, it is striking to note that as societies became more open, death rates, and infection rates trended toward an increase. Analysis using the full set of over 200 nations impacted by the pandemic may enable greater insight into this phenomenon, however, the likely mechanism for the trend is the ability to quickly and efficiently enact public health measures, and a relationship between relatively closed societies and central planning. Analysis of the “authoritarian advantage” in response to the SARS pandemic in China and Taiwan is suggestive of this explanation (44), where the authors suggested that (absence of) centralised decision-making powers, as well as public support and a close relationship (or control) over mass media as reasons why Taiwan was relatively ineffective in combatting SARS in the 2002–2003 outbreak. It is theoretically possible to de-couple an effective public health response from repression of freedom, and countries that have struck a balance between freedom and considered public health response will likely emerge in the coming months.

The most striking finding, however, was the significant *positive* association between national expenditure on healthcare and COVID-19 fatalities. The proportion of healthcare expenditure did not insulate nations from negative COVID-19 outcomes. There are a number of possible explanations for this. Firstly, the total health expenditure budget includes a number of sub-categories, and nations vary widely in the degree to which they spend on research and public health measures, for example. Health care capacity or functionality from the perspective of an infectious disease may be sensitive to decisions about allocation well in advance of the pandemic. Developing nations spend a far greater proportion of their total health budgets on public health measures, and as they develop, their relative spending on public health measures begins to ease. In Bangladesh, for example, in the 10 years to 2007, the nation's proportional expenditure on medicines and medical goods almost tripled, and it's public health and prevention spending fell by 22% (16) while during that same period public health spending in key OECD economies remained static or grew slightly (45). Communicable disease prevention is a relatively lower priority for developed nations, where communicable disease still accounts for 60% of total disease burden, around triple that of the developed world (46). The developed world has greater expertise and relatively greater resources at a grass-roots level to respond to a pandemic. Countries with larger relative and absolute health budgets are also those with proportionally larger populations over 65 presenting with comorbidities, two known risk-factors for COVID-19 mortality. Countries with larger health budgets are also likely able to track cases and deaths more accurately (as % of GDP). This latter explanation seems particularly plausible, and indeed a *post-hoc* analysis demonstrated that countries with a higher healthcare budget also performed more tests per million. However, excess death analyses emerging during this crisis show that even in developed countries reported figures used in the current analysis likely greatly *underestimate* actual deaths associated with COVID-19 (47). We also checked for multicollinearity between healthcare expenditure and capacity using the variance inflation factor (VIF) method, and found it to be non-significant. Finally, health funding in the developing world may be linked to greater oversight by funding bodies, seeking demonstrable value for money, although the success of these aspirations and audit processes in delivering higher impact and value is debatable (48).

Our analysis suggests another explanation: we present evidence of an association between ‘civil society' variables and socioeconomic well-being (49), and our analysis supports the notion that nations that spent more on health were also those nations that afforded their citizens greater freedoms and showed more respect to civil institutions, inadvertently impairing or delaying implementation of public health interventions. In this sense, this adds to an emerging understanding of how societal and cultural factors may be predictive of infection patterns (49).

There are clear disclaimers that must apply to studies conducted in the midst of pandemic. As noted, the number of deaths and confirms cases could be systematically or non-systematically underreported. The data on the number of tests conducted were missing for a number of countries, which did not allow inclusion of testing as a covariate in the regression analysis, but we still used this variable in our *post-hoc* analysis to shed some light on this. Although we had the data on number of hospital beds, another important piece of information about the strength of the healthcare system, country-wise numbers of intensive care unit (ICU) beds, was unavailable. Nurse numbers included a cohort of midwives, with less direct relevance to COVID-19 care. The “civil society” variable used for this study is multidimensional and includes subjective elements that may limit its value to a public health study. Finally, the inclusion criterion (1,000+ cases) may have systematically eliminated countries under-utilising tests to establish prevalence (either through lack of resources or for other reasons). This criterion certainly limited the power of the analysis by excluding over half of the nations with COVID-19 cases. However, it also plausibly eliminated countries with little testing capacity, and thus may improve the validity of the modelling.

The strength of this research in taking advantage of global, recent, and publicly-available data, is also its weakness. The pandemic has evolved so rapidly, that the data represents multiple levels of uncertainty: uncertainty in the numbers themselves (due to testing protocols of COVID-19 being in their relative infancy), and uncertainty in healthcare response. However, this study provides a basis to continue to monitor and update the analyses as the pandemic evolves and data accumulates. In addition, data on a number of factors such as physical activity, smoking prevalence, prevalence of different types of non-communicable disease, behaviour, and travel history are not available which may have an impact on case fatality. Equally importantly, the issue of *access* to health care is not addressed. As appropriate data become available, future studies will be able to take these variables into account.

Just as researchers operating under conditions of great uncertainty, policy-makers and are also forced to deal with incomplete knowledge. Not surprisingly, co-ordination between nations as well as within nations appears to be sub-optimal. This study suggests that population density, underlying burden of NCDs, and low health expenditure on their own may not be as strong predictors of COVID-19 fatality as some current commentary suggests. In suggesting that building effective multi-factorial healthcare capacity (which the current analysis shows is also associated with testing capacity) is an efficient means of mitigating case fatalities in the current pandemic, this study suggests a path forward to responding to COVID-19 and future pandemics: building healthcare capacity focused at a human resource level (nurses as well as doctors), and ensuring adequate hospital beds in reserve. This study confirms previous work showing that these variables are closely but not absolutely correlated with healthcare expenditure (50). Long-term investment in healthcare resources well in advance of pandemics is clearly required, but this study also suggests that government control has a role in significantly reducing the impact of a pandemic.

## Data Availability Statement

Publicly available datasets were analyzed in this study. This data can be found here: John Hopkins University database (https://github.com/CSSEGISandData/COVID-19), World Bank records (https://data.worldbank.org/indicator/), CIVICUS Civil Society Index (https://monitor.civicus.org/).

## Ethics Statement

Ethical review and approval was not required for the study on human participants in accordance with the local legislation and institutional requirements. Written informed consent for participation was not required for this study in accordance with the national legislation and the institutional requirements.

## Author Contributions

JK, NA, and OM conceptualised the study. JK and NA designed the analysis plan. JK performed formal analysis and made the figures. OM, JK, NA, and MI contributed to writing. All authors approved the final version for submission.

## Conflict of Interest

The authors declare that the research was conducted in the absence of any commercial or financial relationships that could be construed as a potential conflict of interest.
