# Supplementary material for: Healthcare Capacity, Health Expenditure, and Civil Society as Predictors of COVID-19 Case Fatalities: A Global Analysis
Source: Front Public Health. 2020 Jul 3;8:347. doi: 10.3389/fpubh.2020.00347 (PMC7349997; doi:10.3389/fpubh.2020.00347)
Supplement: Supplementary file 1 [file Data_Sheet_1.docx]

Appendix 1. List of countries in different healthcare capacity groups.

| country | Healthcare capacity | country | Healthcare capacity | country | Healthcare capacity |
| --- | --- | --- | --- | --- | --- |
| Afghanistan | Low | United Arab Emirates | Medium | Australia | High |
| Bangladesh | Low | Argentina | Medium | Austria | High |
| Bahrain | Low | Armenia | Medium | Belgium | High |
| Bolivia | Low | Azerbaijan | Medium | Bulgaria | High |
| Chile | Low | Bosnia and Herzegovina | Medium | Belarus | High |
| Cote d'Ivoire | Low | Brazil | Medium | Switzerland | High |
| Cameroon | Low | Canada | Medium | Cuba | High |
| Colombia | Low | China | Medium | Czechia | High |
| Djibouti | Low | Spain | Medium | Germany | High |
| Dominican Republic | Low | Estonia | Medium | Denmark | High |
| Algeria | Low | United Kingdom | Medium | Finland | High |
| Ecuador | Low | Greece | Medium | France | High |
| Egypt | Low | Israel | Medium | Croatia | High |
| Ghana | Low | Italy | Medium | Hungary | High |
| Guinea | Low | Kuwait | Medium | Ireland | High |
| Indonesia | Low | Moldova | Medium | Iceland | High |
| India | Low | Mexico | Medium | Japan | High |
| Iran | Low | North Macedonia | Medium | Kazakhstan | High |
| Iraq | Low | New Zealand | Medium | Korea, South | High |
| Morocco | Low | Oman | Medium | Lithuania | High |
| Malaysia | Low | Poland | Medium | Luxembourg | High |
| Nigeria | Low | Portugal | Medium | Netherlands | High |
| Pakistan | Low | Romania | Medium | Norway | High |
| Panama | Low | Saudi Arabia | Medium | Russia | High |
| Peru | Low | Singapore | Medium | Slovakia | High |
| Philippines | Low | Serbia | Medium | Slovenia | High |
| Qatar | Low | Turkey | Medium | Sweden | High |
| Thailand | Low | US | Medium | Ukraine | High |
| South Africa | Low |  |  | Uzbekistan | High |
